# An Optical Fibre Depth (Pressure) Sensor for Remote Operated Vehicles in Underwater Applications

**DOI:** 10.3390/s17020406

**Published:** 2017-02-19

**Authors:** Dinesh Babu Duraibabu, Sven Poeggel, Edin Omerdic, Romano Capocci, Elfed Lewis, Thomas Newe, Gabriel Leen, Daniel Toal, Gerard Dooly

**Affiliations:** 1 Optical Fibre Sensors Research Centre, University of Limerick, Limerick V94 T9PX, Ireland; Sven.Poeggel@ul.ie (S.P.); Thomas.Newe@ul.ie (T.N.); Gabriel.Leen@ul.ie (G.L.); 2 Mobile & Marine Robotics Research Centre, University of Limerick, Limerick V94 T9PX, Ireland; Edin.omerdic@ul.ie (E.O.); Romano.Capocci@ul.ie (R.C.); Daniel.Toal@ul.ie (D.T.); Gerard.Dooly@ul.ie (G.D.)

**Keywords:** ROV, OFPTS, Pressure, sensors, ROV control, Fabry-Perot, Fibre Bragg Gratings

## Abstract

A miniature sensor for accurate measurement of pressure (depth) with temperature compensation in the ocean environment is described. The sensor is based on an optical fibre Extrinsic Fabry-Perot interferometer (EFPI) combined with a Fibre Bragg Grating (FBG). The EFPI provides pressure measurements while the Fibre Bragg Grating (FBG) provides temperature measurements. The sensor is mechanically robust, corrosion-resistant and suitable for use in underwater applications. The combined pressure and temperature sensor system was mounted on-board a mini remotely operated underwater vehicle (ROV) in order to monitor the pressure changes at various depths. The reflected optical spectrum from the sensor was monitored online and a pressure or temperature change caused a corresponding observable shift in the received optical spectrum. The sensor exhibited excellent stability when measured over a 2 h period underwater and its performance is compared with a commercially available reference sensor also mounted on the ROV. The measurements illustrates that the EFPI/FBG sensor is more accurate for depth measurements (depth of ~0.020 m).

## 1. Introduction

Optical fibres have been widely used across a multitude of sensing applications [1]. Typical measurands of optical fibres include pressure and temperature [2,3,4,5,6] with significant benefits in the form of small size, immunity to electromagnetic interference, high sensitivity and low cost [7]. Furthermore, many application areas have emerged, including biomedical, aerospace and marine [8,9,10,11], where pressure/temperature sensors are required at a specific location. Optical fibre based sensors can be utilized in either point or quasi-distributed configurations.

Fibre optic technology is often applied in the area of ocean science in the form of sensors and communications and it is becoming increasingly essential to predict various ocean parameters in the field of climate change, weather forecasts, energy supply, offshore mining and oil industries [12]. High resolution in-situ sensors which can be placed on buoys, moorings and Unmanned Underwater Vehicles (UUVs) can be of major benefit in the monitoring of ocean parameters, e.g., ocean status (pressure fluctuations), depth, temperature, salinity, etc. The environmental parameter changes which need to be recorded are generally very small and existing sensors which have been mounted on floats have to be frequently calibrated to achieve the required accuracy [13]. Optical fibre sensors have several advantages over existing sensor technologies (quartz, inductive or capacitive) [14], e.g., include multiplexing capability, linearity, stability, range (1000 m) and accuracy (+/−% of the depth).

A miniature sensor, based optical fibre technology, is described herein for high resolution measurement of pressure (depth) with temperature compensation for the ocean environment. The sensor, based on an optical fibre Fabry Perot interferometer (EFPI) approach, often used in a number of sensing applications such as temperature, acoustic wave sensing, pressure and liquid level sensing [15,16] will be used to measure pressure underwater. However, EFPI sensors often provide a very high level of sensitivity of single particular physical parameters, but suffer from cross sensitivity, e.g., temperature [17]. To overcome this, it is reported that temperature recorded in-situ can be used to negate the cross-sensitivity issues. The sensor Fibre Bragg Gratings (FBG) is located at very close proximity to the point of measurement. The developed sensor is mechanically robust and was packaged, making it suitable for underwater use. Furthermore, the sensor diaphragm was designed for the specific resolution and range needed for underwater applications. The combined pressure and temperature sensor system was tested in its desired operating environment by mounting it on-board a miniature remotely operated vehicle (ROV) and recording data whilst transitioning through varying water depths. The reflected optical spectrum of the sensor was monitored online and a pressure change caused a corresponding observable shift in the spectrum.

## 2. Sensor Theory and Design

Several pressure sensors based on EFPI sensors have been reported in recent years [17,18,19,20]. The sensor described herein is fabricated entirely from silica glass and the EFPI sensor operation and fabrication technique is discussed in the following section.

### 2.1. Operating Principle of EFPI and FBG

**Figure 1:**
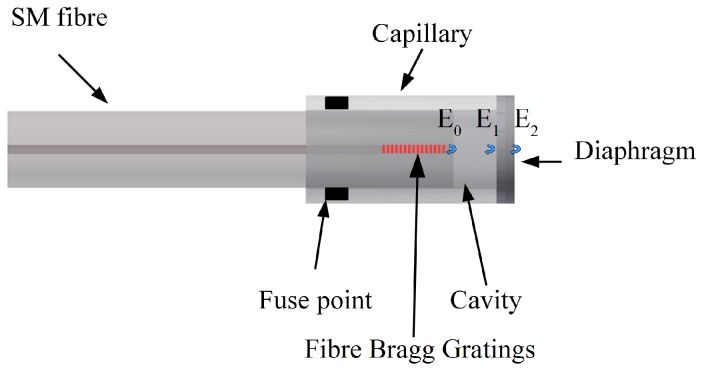
Schematic diagram of the Extrinsic Fabry-Perot interferometer (EFPI) sensor.

The schematic diagram of the EFPI sensor is shown in Figure 1 below. The incident light propagating through the single mode (SM) fibre and is reflected at the glass/air interface of the SM fibre, at the air/glass interface and at the glass/water interfaces of the diaphragm. The reflections at these interfaces transmit back along the SM fibre and form an inference pattern. The phase difference between the incident and the reflected light forms the interference which depends on the cavity length. The relationship between the multiple interference and the back reflection spectrum is expressed in Equation (1) below.

(1) $$I={\left(\vec{E_{0}}+\vec{E_{1}}+\vec{E_{2}}\right)}^{2}$$

(2) $$I\left(\lambda \right)=E_{0}\;\cdot \;E_{1}\;\cdot \;\cos\left(\frac{4\pi n_{0}L}{\lambda }\right)+E_{0}\;\cdot \;E_{2}\;\cdot \;\cos\left(\frac{4\pi \left(n_{0}L+n_{1}d\right)}{\lambda }\right)+E_{1}\;\cdot \;E_{2}\;\cdot \;\cos\left(\frac{4\pi dn_{1}}{\lambda }\right)$$

where ’$E_{0}$’ is the light reflected at the end-face of SM fibre, ’$E_{1}$’ is the light reflected at inner side of the diaphragm(MM), ’$E_{2}$’ is the light reflected at the outer side of the diaphragm, ’*L*’ is the length of the cavity, ’$n_{0}$’, ’$n_{1}$’ are the refractive indices of the air and diaphragm respectively, ’*d*’ thickness of the diaphragm and ’*λ*’ the wavelength. The amplitude of the reflected light is determined by the reflections of each interface. Cavity length changes with the deflection of the diaphragm when an external pressure is applied. The change in cavity length ΔL due to the pressure difference may be expressed as in Equation (3) [21].

(3) $$\Delta L=\left(\frac{3(1-v^{2})}{16Ed^{3}}\right)\;\cdot \;a^{4}\;\cdot \;\Delta P=s_{p}\;\cdot \;\Delta P$$

where ’*d*’ is the diaphragm thickness and ’*a*’ radius, ’*υ*’ Poisson ratio, ΔP is the change in pressure, ’$s_{p}$’ is the pressure sensitivity and E is the Young’s modulus. The deflection of the diaphragm depends linearly on the pressure difference, when it is below 30% of the diaphragm thickness. Increasing the diaphragm thickness results in the increase in the pressure range with decreased resolution.

The light propagating through the SM FBG is reflected back if the optical wavelength is equal to the Bragg wavelength $\lambda_{B}$. The temperature sensitivity of the sensor occurs due of the effect on the induced refractive index change and the thermal coefficient of the SM fibre. The Bragg wavelength shifts $\lambda_{B}\left(\Delta T\right)$ due to a change in temperature ΔT may be expressed as in Equation (4) where ’*k*’ and ’$\lambda_{B}\left(T_{0}\right)$’ are the temperature sensitivity and initial wavelength, respectively [22,23].

(4) $$\begin{array}{c} \lambda_{B}\left(\Delta T\right)=\lambda_{B}\left(T_{0}\right)+k\;\cdot \;\Delta T \end{array}$$

The compensation for the cross-sensitivity of thermal effects on the EFPI sensor is achieved by using the matrix where the temperature sensitivity $s_{t}$ is determined in advance [24].

(5) $$\left[\begin{array}{c} {\Delta \lambda }_{B}\\ \Delta L \end{array}\right]=\left[\begin{array}{cc} 0 & k\\ s_{p} & s_{t} \end{array}\right]\left[\begin{array}{c} \Delta P\\ \Delta T \end{array}\right]$$

### 2.2. Fabrication of Optical Fibre Pressure and Temperature Sensor (OFPTS)

**Figure 2:**
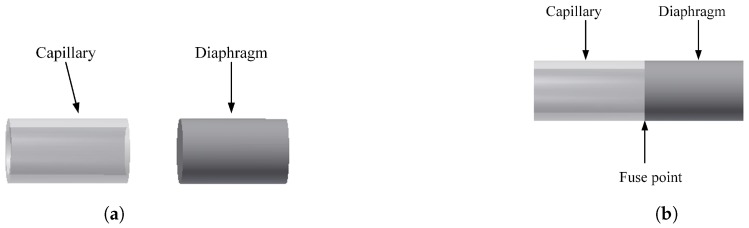

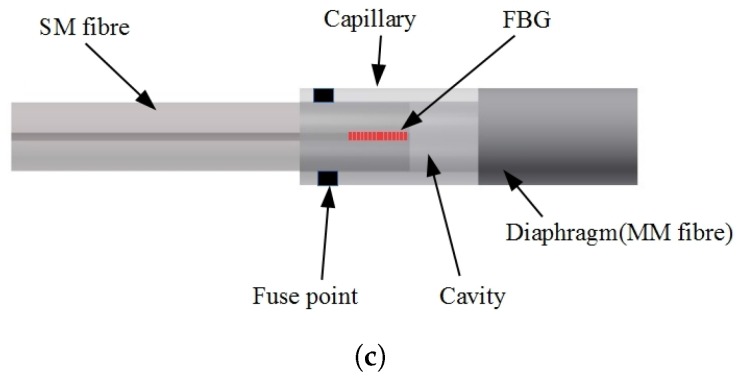
Sensor fabrication process: (**a**) The Capillary and the diaphragm (MM) fibre was polished (**b**) The polished fibres are fused together (**c**) The single mode fiber with Fibre Bragg Grating (FBG) is inserted into the capillary and fused with a air cavity.

The sensor fabrication process is shown schematically in Figure 2. The EFPI pressure sensor is fabricated by splicing the polished capillary (outer diameter 200 *μ*m) and a multimode (MM) fibre (with a diameter of 200 *μ*m), which forms a diaphragm. The single mode (SM) fibre with the Fibre Bragg Gratings from FBGS Technologies GMBH is cleaved and inserted into the capillary.

**Figure 3:**
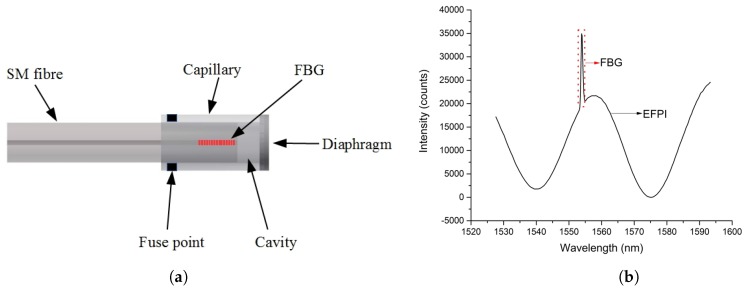
(**a**) Schematic of the EFPI/FBG sensor; (**b**) Reflection spectrum of the sensor.

The FBGs is 2 mm in length with a Bragg wavelength of 1554 nm and reflectivity of about 97%. The FBG is completely enclosed inside the capillary and is then spliced to the capillary forming an air cavity of ~20–30 *μ*m using the Ericsson FSU 975 splicer. During splicing process the reflectivity of FBG is reduced. The sensor head is cleaved and polished manually to a desired thickness (10–11 *μ*m) and then further etched chemically using hydrofluoric(HF) acid to reduce the thickness of the diaphragm (3–4 *μ*m). The complete fabrication process is monitored online in real time using a custom built LabVIEW${}^{TM}$ program which permits precise online monitoring of the diameter thickness during the etching stage of the fabrication process. The schematic of the EFPI/FBG sensor and the reflection spectrum is shown in Figure 3.

## 3. Setup for the Measurements

### 3.1. Optical System Setup

**Figure 4:**
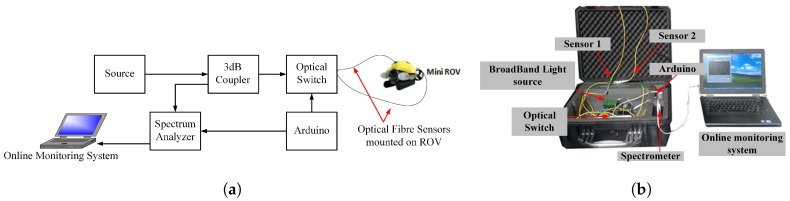
Optical System Setup: (**a**) Schematic of the optical system; (**b**) Optical System setup for using two sensors.

A schematic of the developed sensing systems and photograph are shown in Figure 4a and Figure 4b respectively. The interrogation system consists of broadband light source(superluminescent LED from Exalos EXS210069-01); a 3 dB coupler; an optical switch (Sercalo SW 1x4-9N); an optical spectrum analyser (OSA) (Ibsen I-MON-512E). The broadband light source has a Gaussian spectral shape with an optical power output upto 14.97 mW and with a bandwidth of 46.2 nm at 1554.2 nm is used. The source was connected to a 3-dB optical coupler guiding the light to an optical switch which has a 1 ms switching time. The light is reflected from the sensor and coupled back to the spectrometer (Ibsen Photonics IMON 512E). The spectrometer has a resolution of 512 pixels with a bandwidth from 1510–1595 nm. The optical switch was operated with a frequency of 20 Hz corresponding to an interval of 5 frames/cycle and 2 cycles/sensor resulting in a 10 Hz sample rate for each sensor. An Arduino board was used to control the optical switch with a switching period of 2 ms. The interrogation time was adjusted depending on the sensor and the attenuator was used to balance the optical power of the signal for the two sensors used.

### 3.2. On-Board Measurement System Setup

**Figure 5:**
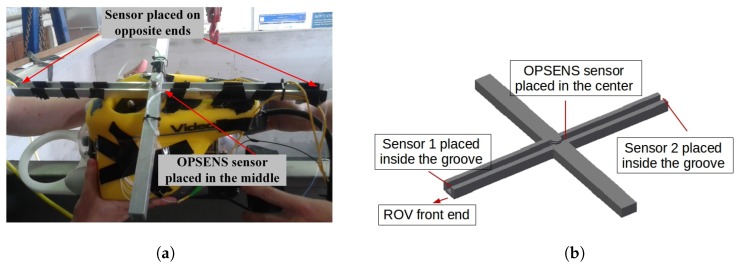
Measurement setup (**a**) Sensors mounted on remotely operated vehicle (ROV), and (**b**) Sensors placement on the mount.

The measurement system mounted on board the ROV is shown photographically in Figure 5a. The developed sensors can be used to accurately monitor the pressure in an ocean or similar environment. However, if two pressure sensors are placed at opposite ends of an ROV the differential reading from both sensors can be used in determining of the ROV depth accurately. The mount was fixed on the ROV so that it can accomodate three sensors, two EFPI optical and one commercially available optical fibre pressure probe (OPSENS OPP-M) sensors as shown in Figure 5b. The OPSENS sensor was placed exactly in the centre of the mount while the EFPI sensors were placed on the ends on either side. The configuration provides critical information on the depth and the ROV’s stability when moving underwater.

## 4. Experimental Results

### 4.1. Calibration of Optical Pressure Sensors

**Figure 6:**
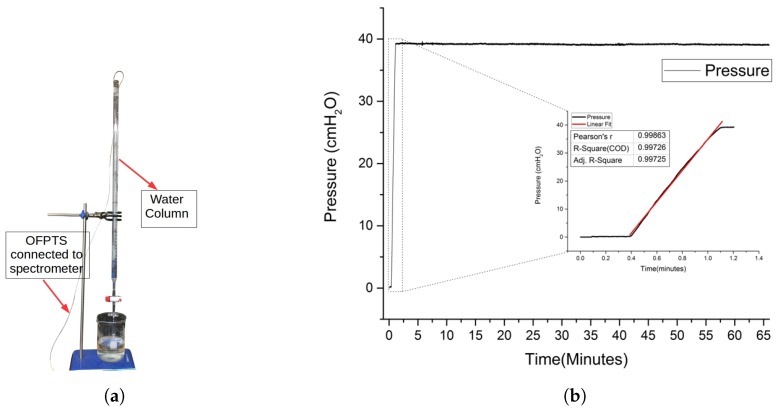
Measurement setup (**a**) Sensor calibration in a watercolumn, and (**b**) Calibration measurement and the stability of the sensor for 1 h.

**Figure 7:**
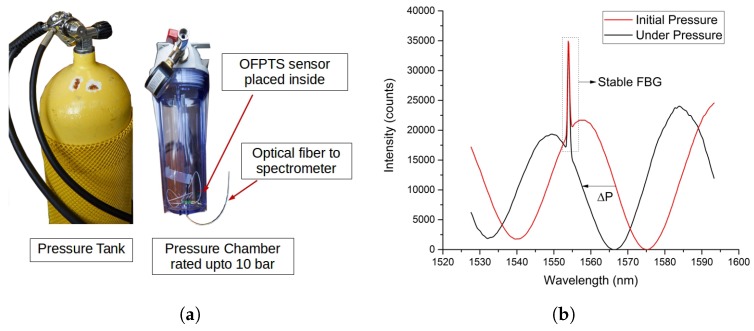
(**a**) Sensor calibration in the pressure chamber and (**b**) EFPI/FBG spectrum with initial and under pressure.

The fabricated sensors were calibrated using a standard water column (Figure 6a) as well as a commercial pressure chamber (Figure 7a) for use with pressures up to 10 bar (~101 *m* depth in water). The pressure gauge (Badotherm BDT18-63MM) with accuracy of 1.6% full scale value (~163 *cm H_2_O*) and pressure range upto 10 bar was used as a reference.

The sensor was also placed in a standard water column and the response for the varying change in pressure. The sensitivity of the sensor was determined as 10–20 *nm/kPa* with a resolution of 0.8 *cm H_2_O*. The sensor was placed inside the high pressure chamber and the pressure was raised gradually, the FPI spectrum shift was observed based on the external pressure applied as shown in Figure 7b. The pressure sensor was therefore further calibrated for a range extending from 0–90 m. The sensor was calibrated for stability and noise for 1 h under 39 *cm H_2_O* pressure as shown in Figure 6b with a noise level less than ~0.5% of the full scale reading.

**Figure 8:**
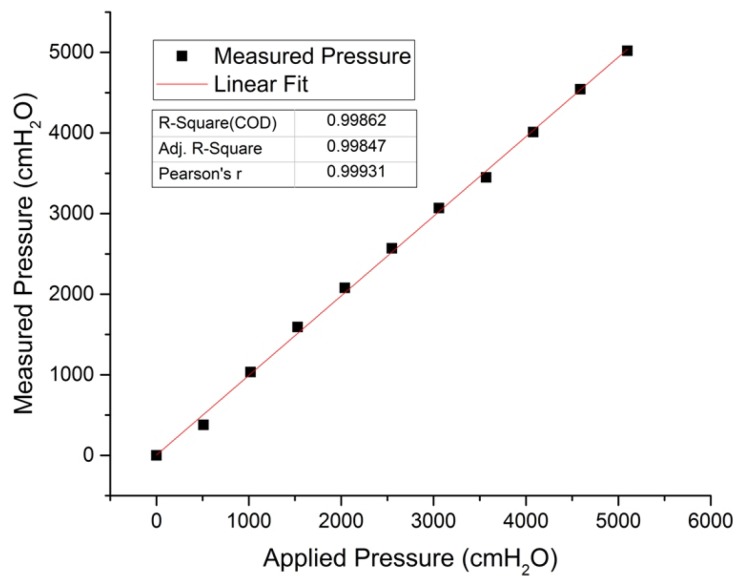
Pressure response of EFPI/FBG sensor for 5 bar.

**Figure 9:**
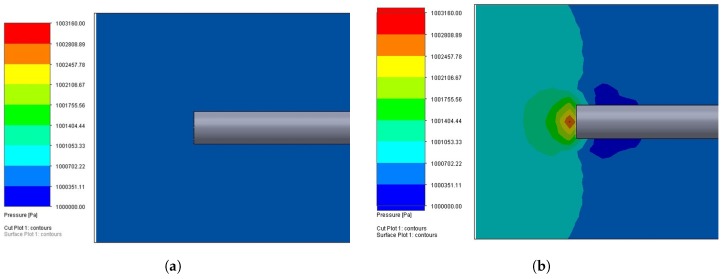
(**a**) Theoretical Simualtion with no flow, and (**b**) Theoretical Simulation with flow of 6 knots (3 m/s) at 10 bar pressure.

The OFPTS sensors exhibits a linear response as shown in Figure 8 when pressure is applied from zero to 5 bar (5098.58 *cm H_2_O*) with R^2^ = 0.99862. The cross sensitivity of the FBG due to pressure is negligible as shown in Figure 7b under pressure since it is completely enclosed inside the capillary. The cross sensitivity of the sensor (at 10 bar) due to water flow was estimated ~0.3% full scale value (~30 *cm H_2_O*) by a theoretical simulation model as shown in Figure 9b with flow of 6 knots(3 *m/s*).

**Figure 10:**
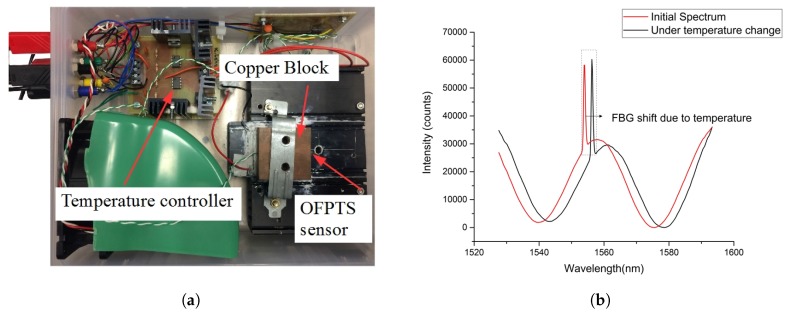
(**a**) Sensor calibration in the temperature chamber; and (**b**) EFPI/FBG spectrum with initial and under temperature.

The OFPTS sensor was calibrated for temperature measurements using a temperature controller block with a Peltier element designed for OFPTS (Figure 10a) and in a temperature chamber which reaches 200 °C. The copper block was mounted on top of the Peltier element and the temperature was monitored using a thermocouple (Z2-K-1M). The reflection spectrum of the sensor is shown in Figure 10b as the temperature was increased from room temperature to 130 °C. The temperature sensitivity of ~12.5 *pm/K* was determined with a resolution of 0.1 °C.

### 4.2. Measurement on ROV

**Figure 11:**
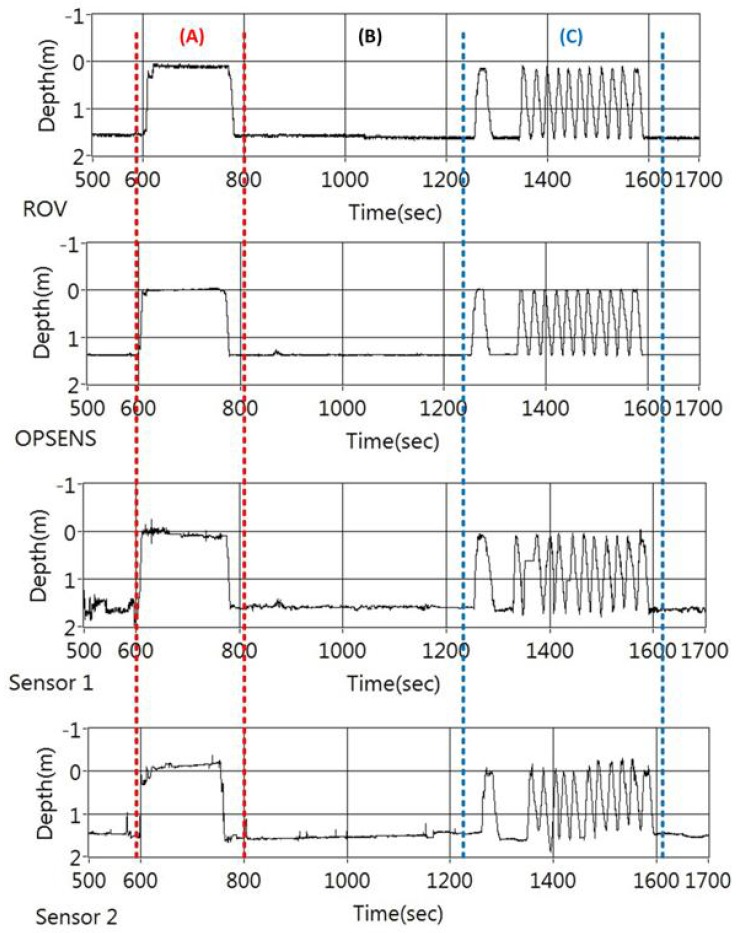
Pressure (depth) response of the sensors mounted on the ROV where section (A) represents the ROV moving slowly to the surface in the tank, section (B) represents the ROV moving slowly in the bottom of the tank and section (C) represents the ROV moving rapidly to the surface and to the bottom of the tank.

The optical fibre sensors of this investigation were packaged in a small tube and mounted on the ROV as shown in Figure 5a. The ROV was controlled from an off-site location and was in motion continuously inside the tank. The sensor was monitored online with a custom built LabVIEW program which was used to capture and analyze all ROV data. The sensors response at varying depths is shown in Figure 11. The OFPTS sensor was measured against the commercial sensor mounted on the ROV and against the OPSENS probe. The OFPTS sensor was found to be more sensitive than the electrical sensor mounted on the ROV and correlates with the OPSENS probe. Figure 11 shows the response of the sensor for varying depths, which is split into three different parts for analysis where section (A) represents the ROV moving slowly to the surface in the tank, section (B) represents the ROV moving slowly in the bottom of the tank and section (C) represents the ROV moving rapidly to the surface and to the bottom of the tank.

**Figure 12:**
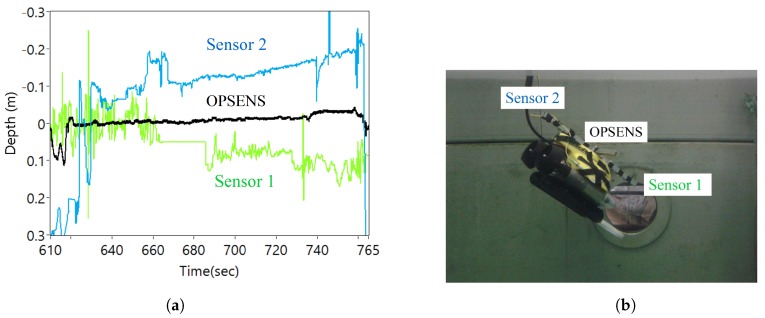
(**a**) Pressure (depth) response of the sensors mounted on the ROV from Figure 11 section (A); and (**b**) Illustration of ROV orientation.

The Figure 12a shows the sensor response of the sensors when the ROV was moved up and down in the tank. The OFPTS sensors 1 and 2 provide different values of the depth (orientation) while data from the OPSENS stays in the middle. The difference in sensors 1 and 2 values is as expected as the ROV was tilted in the horizontal while maintaining a constant depth. Figure 11(C) shows the response of the sensors for repeatability when moved rapidly up and down in identical manoeuvre in short intervals.

**Figure 13:**
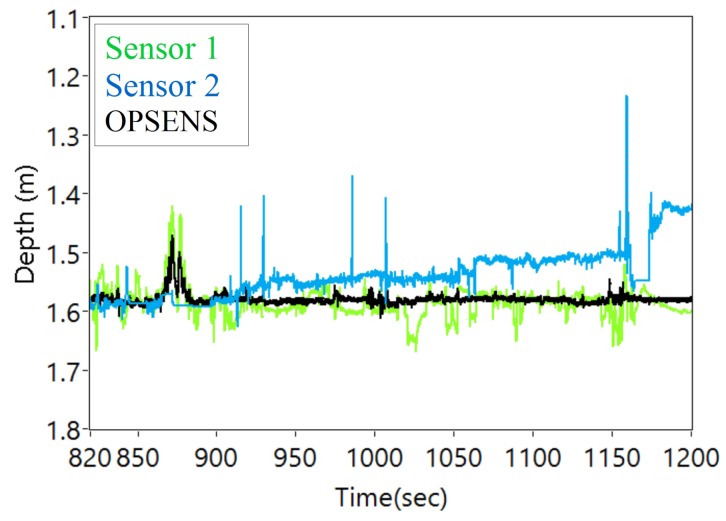
Pressure (depth) response of the sensors at the bottom of the tank when moving continuously from Figure 11(B).

The Figure 13 shows the pressure response of the sensors when at the bottom of the tank when moving continuously. The ROV was slightly tilted due to the umbilical cord during movement which resulted in the small difference in depth between the sensors. The spikes in the sensor 2 was due to the thrusters of the ROV.

## 5. Conclusions

In this paper, the fabrication of two identical miniature optical fibre EFPI based sensors for pressure (depth) measurements is discussed. The OFPTS sensors are based on EFPI and FBG was tested on a small ROV in a lab based water tank for repeatability, resolution and accuracy against electrical commercial sensors also mounted on the ROV. The commercial sensor on the ROV has a depth resolution of only 4 *cm* which is inferior to the two OFPTS sensors. The investigation of this paper has shown the two OFPTS sensors to have a resolution of 0.8 *cm H_2_O* and an accuracy of 2 *cm H_2_O*. This work has demonstrated that the miniature OFPTS sensors can be utilised in the marine environment as the full interrogation system can be completely housed in the marinised pressure-resistant component of a typical ROV.

## Abbreviations

The following abbreviations are used in this manuscript:

EFPI	Extrinsic Fabry-Perot Interferometer
FBG	Fibre Bragg Gratings
HF	HydroFluoric Acid
MM	Multi Mode
NI	National Instruments
OFPTS	Optical Fiber Pressure and Temperature Sensor
OSA	Optical Spectrum Analyzer
ROV	Remote Operated Vehicle
SM	Single Mode
UUV	Unmanned Underwater Vehicles
